# Change-Point Detection Using the Conditional Entropy of Ordinal Patterns

**DOI:** 10.3390/e20090709

**Published:** 2018-09-14

**Authors:** Anton M. Unakafov, Karsten Keller

**Affiliations:** 1 Institute of Mathematics, University of Lübeck, 23562 Lübeck, Germany; anton@math.uni-luebeck.de or; 2 Graduate School for Computing in Medicine and Life Sciences, University of Lübeck, 23562 Lübeck, Germany; 3 Georg-Elias-Müller-Institute of Psychology, University of Goettingen, Goßlerstraße 14, 37073 Goettingen, Germany; 4 Theoretical Neurophysics Group, Max Planck Institute for Dynamics and Self-Organization, Am Fassberg 17, 37077 Goettingen, Germany; 5 Leibniz ScienceCampus Primate Cognition, Kellnerweg 4, 37077 Goettingen, Germany

**Keywords:** change-point detection, conditional entropy, ordinal pattern

## Abstract

This paper is devoted to change-point detection using only the ordinal structure of a time series. A statistic based on the conditional entropy of ordinal patterns characterizing the local up and down in a time series is introduced and investigated. The statistic requires only minimal a priori information on given data and shows good performance in numerical experiments. By the nature of ordinal patterns, the proposed method does not detect pure level changes but changes in the intrinsic pattern structure of a time series and so it could be interesting in combination with other methods.

## 1. Introduction

Most of real-world time series are non-stationary, that is, some of their properties change over time. A model for some non-stationary time series is provided by a piecewise stationary stochastic process: its properties are locally constant except for certain time-points called *change-points*, where some properties change abruptly [1].

Detecting change-points is a classical problem being relevant in many applications, for instance in seismology [2], economics [3], marine biology [4], and in many other science fields. There are many methods for tackling the problem [1,5,6,7,8]. However, most of the existing methods have a common drawback: they require certain a priori information about the time series. It is necessary to know either a family of stochastic processes providing a model for the time series (see for instance [9] where autoregressive (AR) processes are considered) or at least to know which characteristics (mean, standard deviation, etc.) of the time series reflect the change (see [7,10]). In real-world applications, such information is often unavailable [11].

Here, we suggest a new method for change-point detection that requires minimal a priori knowledge: we only assume that the changes affect the evolution rule linking the past of the process with its future (a formal description of the considered processes is provided by Definition 4). A natural example of such change is an alteration of the increments distribution.

**Figure 1:**
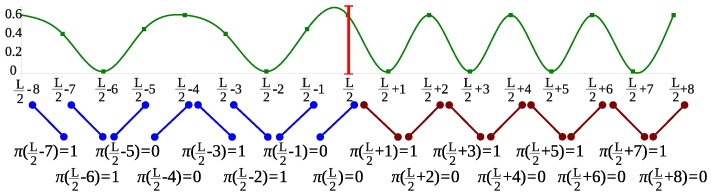
A part of a piecewise stationary time series with a change-point at t=L/2 (marked by a vertical line) and corresponding ordinal patterns of order d=1 (below the plot).

Our method is based on ordinal pattern analysis, a promising approach to real-valued time series analysis [12,13,14,15,16,17,18]. In ordinal pattern analysis, one considers order relations between values of a time series instead of the values themselves. These order relations are coded by ordinal patterns; specifically, an ordinal pattern of an order d∈N describes order relations between (d+1) successive points of a time series. The main step of ordinal pattern analysis is the transformation of an original time series into a sequence of ordinal patterns, which can be considered as an effective kind of discretization extracting structural features from the data. A result of this transformation is demonstrated in Figure 1 for order d=1. Note that the distribution of ordinal patterns contains much information on the original time series making them interesting for data analysis, especially for data from nonlinear systems (see [19,20]).

For detecting a change-point $t^{*}\in N$ in a time series $x={\left(x(t)\right)}_{t=0}^{L}$ with values in R, one generally considers *x* as a realization of a stochastic process *X* and computes for *x* a statistic S(t;x) that should reach its maximum at $t=t^{*}$. Here, we suggest a statistic on the basis of the conditional entropy of ordinal patterns introduced in [21]. The latter is a complexity measure similar to the celebrated permutation entropy [12] with particularly better performance (see [20,21]).

Let us provide an “obvious” example only to motivate our approach and to illustrate its idea.

**Example** **1.** *Consider a time series ${\left(x(t)\right)}_{t=0}^{L}$, and its central part is shown in Figure 1. The time series is periodic before and after L/2, but, at L/2, there occurs a change (marked by a vertical line): the “oscillations” become faster. Figure 1 also presents the ordinal patterns π(t) of order d=1 at times t underlying the time series. Note that there are only two ordinal patterns of order* 1: *the increasing (coded by* 0*) and the decreasing (coded by* 1*). Both ordinal patterns occur with the same frequency before and after the change-point.*

*However, the transitions between successive ordinal patterns change at L/2. Indeed, before the change-point L/2, both ordinal patterns have two possible successors (for instance, the ordinal pattern π(L/2−5)=0 is succeeded by the ordinal pattern π(L/2−4)=0, which in turn is succeeded by the ordinal pattern π(L/2−3)=1), whereas after the change-point the ordinal patterns *0* and *1* are alternating. A measure of diversity of transitions between ordinal patterns is provided by the conditional entropy of ordinal patterns. For the sequence ${\left(\pi (k)\right)}_{k=1}^{L}$ of ordinal patterns of order *1*, the (empirical) conditional entropy for t=2,3,…,L is defined as follows:*

$$\begin{array}{cc} \mathrm{eCE}\left({\left(\pi (k)\right)}_{k=1}^{t}\right)= & -\sum_{i=0}^{1}\sum_{j=0}^{1}\frac{n_{i,j}\left(t\right)}{t-1}\ln\frac{n_{i,j}\left(t\right)}{n_{i}\left(t\right)},\\ \mathrm{with}\;n_{i,j}\left(t\right) & =\#\{l=1,2,\ldots ,t-1|\pi (l)=i,\pi (l+1)=j\}\\ n_{i}\left(t\right) & =\#\{l=1,2,\ldots ,t-1|\pi (l)=i\}, \end{array}$$

*(throughout the paper, 0ln0:=0 and, more general, 0·a:=0 if a term a is not defined, and #A denotes the number of elements of a set A).*

*To detect change-points, we use a test statistic for d=1 defined as follows:*

$$\begin{array}{cc} \mathrm{CEofOP}\left(\theta L\right)=\left(L-2\right)\;\mathrm{eCE}\left({\left(\pi (k)\right)}_{k=1}^{L}\right) & -\left(\theta L-1\right)\;\mathrm{eCE}\left({\left(\pi (k)\right)}_{k=1}^{\theta L}\right)\\ & -\left(L-(\theta L+1)\right)\;\mathrm{eCE}\left({\left(\pi (k)\right)}_{k=\theta L+1}^{L}\right), \end{array}$$

**Figure 2:**
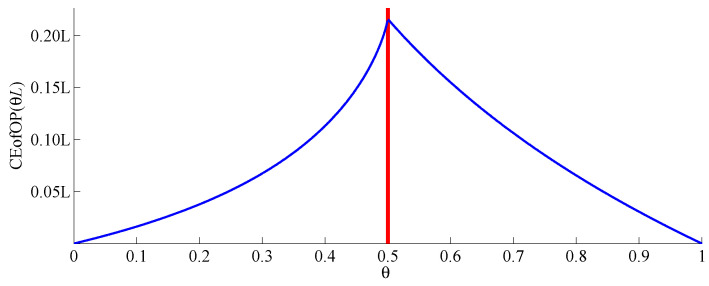
Statistic CEofOP(θL) for the sequence of ordinal patterns of order 1 for the time series from Figure 1.

*for θ∈(0,1) with θL∈N. According to the properties of conditional entropy (see Section 2.2 for details), CEofOP(θL) attains its maximum when θL coincides with a change-point. Figure 2 demonstrates this for the time series from Figure 1.*

For simplicity and in view of real applications, in Example 1, we define ordinal patterns and the CEofOP statistic immediately for concrete time series. However, for theoretical consideration, it is clearly necessary to define the CEofOP statistic for stochastic processes. For this, we refer to Section 2.2.

To illustrate applicability of the CEofOP statistic, let us discuss a real-world data example. Note that here multiple change-points are detected as described below.

**Figure 3:**
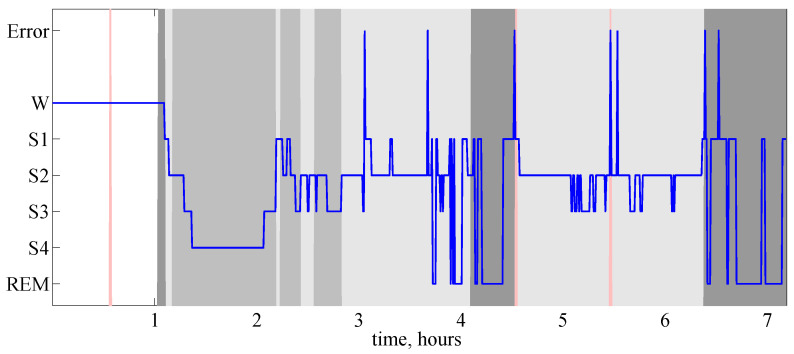
Hypnogram (bold curve) and the results of ordinal-patterns-based discrimination of sleep EEG. Here, the y-axis represents the results of the expert classification: W stands for waking, stages S1, S2 and S3, S4 indicate light and deep sleep, respectively, REM stands for REM sleep and Error—for unclassified samples. Results of ordinal-patterns-based discrimination are represented by the background colour: white colour indicates epochs classified as waking state, light gray—as light sleep, gray—as deep sleep, dark gray—as REM, red colour indicates unclassified segments

**Example** **2.** *Here, we consider electroencephalogram (EEG) recording 14 from the sleep EEG dataset kindly provided by Vasil Kolev (see Section 5.3.2 in [22] for details and further results on this dataset). We employ the following procedure for an automatic discrimination between sleep stages from the EEG time series: first, we split time series into pseudo-stationary intervals by finding change-points with the CEofOP statistic (change-points are detected in each EEG channel separately), then we cluster all the obtained intervals. Figure 3 illustrates the outcome of the proposed discrimination for single EEG channel in comparison with the manual scoring by an expert; the automated identification of a sleep type (waking, REM, light sleep, deep sleep) is correct for 79.6% of 30-s epochs. Note that the borders of the segments (that is the detected change-points) in most cases correspond to the changes of sleep stage.*

The CEofOP statistic was first introduced in [18], where we have employed it as a component of a method for sleep EEG discrimination. However, no theoretical details of the method for change-point detection were provided there. This paper aims to fill in this gap and provides a justification for the CEofOP statistic. Numerical experiments given in the paper show better performance of our method than of a similar one based on the Corrected Maximum Mean Discrepancy (CMMD) statistic developed by one of the authors and collaborators [23,24]. A numerical comparison with the classical parametric Brodsky–Darkhovsky method [11] suggests good applicability of the method to nonlinear data, in particular if there is no level change. This is remarkable since our method is only based on the ordinal structure of a time series.

Matlab 2016 (MathWorks, Natick, MA, USA) scripts implementing the suggested method are available at [25].

## 2. Methods

This section is organized as follows. In Section 2.1, we provide a brief introduction into ordinal pattern analysis. In particular, we define the conditional entropy of ordinal patterns and discuss its properties. In Section 2.2, we introduce the CEofOP statistic. In Section 2.3, we formulate an algorithm for detecting multiple change-points by means of the CEofOP statistic.

### 2.1. Preliminaries

Central objects of the following are stochastic processes $X={\left(X(t)\right)}_{t=n}^{m}$ on a probability space (Ω,A,P) with values in R. Here, $n\in N_{0}$ and n<m∈N∪{∞}, allowing both finite and infinite lengths of processes. We consider only univariate stochastic processes to keep notation simple, however—with the appropriate adaptations—there are no principal restrictions on the dimension of a process. $X={\left(X(t)\right)}_{t=n}^{m}$ is *stationary* if, for all $t_{1},t_{2},\ldots ,t_{k},s$ with $t_{1},t_{2},\ldots ,t_{k},t_{1}+s,t_{2}+s,\ldots ,t_{k}+s\in \left\{n,n+1,\ldots ,m\right\}$, the distributions of ${\left(X_{t_{i}}\right)}_{i=1}^{k}$ and ${\left(X_{t_{i}+s}\right)}_{i=1}^{k}$ coincide.

Throughout this paper, we discuss detection of change-points in a piecewise stationary stochastic process. Simply speaking, a piecewise stationary stochastic process is obtained by “gluing” several pieces of stationary stochastic processes (for a formal definition of piecewise stationarity, see, for instance, ([26], Section 3.1)).

In this section, we recall the basic facts from ordinal pattern analysis (Section 2.1.1), present the idea of ordinal-patterns-based change-point detection (Section 2.1.2), and define the conditional entropy of ordinal patterns (Section 2.1.3).

#### 2.1.1. Ordinal Patterns

Let us recall the definition of an ordinal pattern [14,17,18].

**Definition** **1.** *For d∈N, denote the set of permutations of {0,1,…,d} by $S_{d}$. We say that a real vector $\left(x_{0},x_{1},\ldots ,x_{d}\right)$ has ordinal pattern $\mathrm{OP}\left(x_{0},x_{1},\ldots ,x_{d}\right)=\left(r_{0},r_{1},\ldots ,r_{d}\right)\in S_{d}$ of order d∈N if*

$$x_{r_{0}}\geq x_{r_{1}}\geq \ldots \geq x_{r_{d}}$$

*and*

$$r_{l-1}>r_{l}\;\mathrm{for}\;x_{r_{l-1}}=x_{r_{l}}.$$

As one can see, there are (d+1)! different ordinal patterns of order *d*.

**Definition** **2.** *Given a stochastic process $X={\left(X(t)\right)}_{t=0}^{L}$ for L∈N∪{∞}, the sequence $\Pi^{d}={\left(\Pi (t)\right)}_{t=d}^{L}$ with*

$$\Pi \left(t\right)=\mathrm{OP}\left(X(t-d),X(t-d+1),\ldots ,X(t)\right)$$

*is called the random sequence of ordinal patterns of order d∈N of the process X. Similarly, given $x={\left(x\left(t\right)\right)}_{t=0}^{L}$ a realization of X, the sequence of ordinal patterns of order d for x is defined as $\pi^{d,L}={\left(\pi (t)\right)}_{t=d}^{L}$ with*

$$\pi \left(t\right)=\mathrm{OP}\left(x(t-d),x(t-d+1),\ldots ,x(t)\right).$$

*For simplicity, we say that L∈N is the length of the sequence $\pi^{d,L}$; however, in fact, it consists of (L−d+1) elements.*

**Definition** **3.** *A stochastic process $X={\left(X(t)\right)}_{t=0}^{L}$ for L∈N∪{∞} is said to be ordinal-d-stationary if for all $i\in S_{d}$ the probability $P\left(\Pi (t)=i\right)$ does not depend on t for d≤t≤L. In this case, we call*

(1) $$p_{i}=P\left(\Pi (t)=i\right)$$

*the probability of the ordinal pattern $i\in S_{d}$ in X.*

The idea of ordinal pattern analysis is to consider the sequence of ordinal patterns and the ordinal patterns distribution obtained from it instead of the original time series. Though implying the loss of nearly all the metric information, this often allows for extracting some relevant information from a time series, in particular, when it comes from a complex system. For example, ordinal pattern analysis provides estimators of the Kolmogorov–Sinai entropy [21,27,28] of dynamical systems, measures of time series complexity [12,18,29], measures of coupling between time series [16,30] and estimators of parameters of stochastic processes [13,31] (see also [15,32] for a review of applications to real-world time series). Methods of ordinal pattern analysis are invariant with respect to strictly-monotone distortions of time series [14] do not need information about range of measurements, and are computationally simple [17]. This qualifies it for application in the case that no much is known about the system behind a time series, possibly as a first exploration step.

For a discussion of the properties of ordinal patterns sequence, we refer to [13,31,33,34,35]. For the following, we need two results stated below.

**Lemma** **1** (Corollary 2 from [33])**.** *Each process $X={\left(X(t)\right)}_{t\in N_{0}}$ with associated stationary increment process ${\left(X\left(t\right)-X\left(t-1\right)\right)}_{t\in N}$ is ordinal-d-stationary for each d∈N.*

Probability distributions of ordinal patterns are known only for some special cases of stochastic processes [13,33,35]. In general, one estimates probabilities of ordinal patterns by their empirical probabilities. Consider a sequence $\pi^{d,L}$ of ordinal patterns. For any t∈{d+1,d+2,…,L}, the frequency of occurrence of an ordinal pattern $i\in S_{d}$ among the first (t−d) ordinal patterns of the sequence is given by

(2) $$n_{i}\left(t\right)=\#\left\{l\in \left\{d,d+1,\ldots ,t-1\right\}|\pi \left(l\right)=i\right\}.$$

Note that, in Equation (2), we do not count π(l) with l=t in order to be consistent with the conditional entropy following below and considering two successive ordinal patterns. A natural estimator of the probability of an ordinal pattern *i* in the ordinal-*d*-stationary case is provided by its relative frequency in the sequence $\pi^{d,L}$:

$${\hat{p}}_{i}=\frac{n_{i}\left(L\right)}{L-d}.$$

#### 2.1.2. Stochastic Processes with Ordinal Change-Points

Sequences of ordinal patterns are invariant to certain changes in the original stochastic process *X*, such as shifts (adding a constant to the process) ([15], Section 3.4.3) and scaling (multiplying the process by a positive constant) [14]. However, in many cases, changes in the original process *X* affect also the corresponding random sequences of ordinal patterns and ordinal patterns distributions. On the one hand, this impedes application of ordinal pattern analysis to non-stationary time series. Namely, most of ordinal-patterns-based quantities require ordinal-*d*-stationarity of a time series [12,15,16] and may be unreliable when this condition fails. On the other hand, one often can detect change-points in the original process by detecting changes in the sequence of ordinal patterns.

Below, we consider piecewise stationary stochastic processes that are processes consisting of several stationary segments glued together. The time points where the signals are glued correspond to abrupt changes in the properties of the process and are called *change-points*. The first ideas of using ordinal patterns for detecting change-points were formulated in [23,24,34,36,37,38]. The advantage of the ordinal-patterns-based methods is that they require less information than most of the existing methods for change-point detection: it is assumed that the stochastic process is not from a specific family and that the change does not affect specific characteristics of the process. Instead, we consider further change-points with the following property.

**Definition** **4.** Let ${\left(X(t)\right)}_{t=0}^{L}$ with L∈N∪{∞} be a piecewise stationary stochastic process with a change-point $t^{*}\in N$. We say that $t^{*}$ is an ordinal change-point if there exist some m,n∈N with $m<t^{*}<n\leq L$ and some d∈N such that ${\left(X(t)\right)}_{t=m}^{t^{*}}$ and ${\left(X(t)\right)}_{t=t^{*}+1}^{n}$ are ordinal-d-stationary but ${\left(X(t)\right)}_{t=m}^{n}$ is not. A stochastic process of length less than d+1 is ordinal-d-stationary by definition.

This approach seems to be natural for many stochastic processes and real-world time series. Note that a change-point where a change in mean occurs need not be ordinal, since the mean is irrelevant for the distribution of ordinal patterns ([15], Section 3.4.3). However, there are many methods that effectively detect changes in mean; the proposed method here is intended for use in a more complex case, when there is no classical method, or it is not clear, which of them to apply.

We illustrate Definition 4 by two examples. Piecewise stationary autoregressive processes considered in Example 3 are classical and provide models for linear time series. Since many real-world time series are nonlinear, we introduce in Example 4 a process originated from nonlinear dynamical systems. These two types of processes are used throughout the paper for empirical investigation of change-point detection methods.

**Example** **3.** A first order piecewise stationary autoregressive (AR) process with change-points $t_{1}^{*},t_{2}^{*},\ldots ,t_{N_{\mathrm{st}}-1}^{*}$ is defined as

$$\mathrm{AR}\left(\left(\phi_{1},\phi_{2},\ldots ,\phi_{N_{\mathrm{st}}}\right),\left(t_{1}^{*},t_{2}^{*},\ldots ,t_{N_{\mathrm{st}}-1}^{*}\right)\right)={\left(\mathrm{AR}(t)\right)}_{t=0}^{L},$$

**Figure 4:**
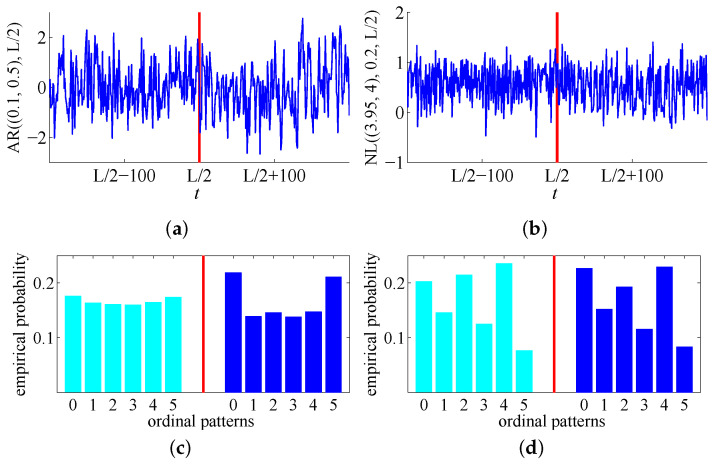
Upper row: parts of realizations of piecewise stationary autoregressive (AR) (**a**) and noisy logistic (NL) (**b**) processes with change-points marked by vertical lines, *L* = 20,000. Lower row: empirical probability distributions of ordinal patterns of order d=2 in the realizations of AR (**c**) and NL (**d**) processes are different before and after the change-point.

*where $\phi_{1},\phi_{2},\ldots ,\phi_{N_{\mathrm{st}}}\in \left[0,1\right)$ are the parameters of the autoregressive model and*

$$\mathrm{AR}\left(t\right)=\phi_{k}\mathrm{AR}\left(t-1\right)+\epsilon \left(t\right),$$

for all $t\in \{t_{k-1}^{*}+1,t_{k-1}^{*}+2,\ldots ,t_{k}^{*}\}$ for $k=1,2,\ldots ,N_{\mathrm{st}}$, where $t_{0}^{*}:=0$ and $t_{N_{\mathrm{st}}}^{*}:=L$, with ϵ being the standard white Gaussian noise, and AR(0): = ϵ(0). AR processes are often used for the investigation of methods for change-points detection (see, for instance, [23,24]), since they provide models for a wide range of real-world time series. Figure 4a illustrates a realization of a ‘two piece’ AR process with a change-point at L/2. By ([13], Proposition 5.3), the distributions of ordinal patterns of order d≥2 reflect change-points for piecewise stationary AR processes. Figure 4c illustrates this for the realization from Figure 4a: empirical probability distributions of ordinal patterns of order d=2 before and after the change-point L/2 differ considerably.

**Example** **4.** *A classical example of a nonlinear system is provided by the logistic map on the unit interval:*

(3) $$x\left(t\right)=rx\left(t-1\right)\left(1-x(t-1)\right),$$

*with t∈N, for x(0)∈[0,1] and r∈[1,4]. The behaviour of this map significantly varies for different value r; we are especially interested in r∈[3.57,4] with chaotic behaviour. In this case, there exists an invariant ergodic measure absolutely continuous with respect to the Lebesgue measure [39,40], therefore Equation (3) defines a stationary stochastic process ${\mathrm{NL}}_{0}$:*

$${\mathrm{NL}}_{0}\left(t\right)=r\left(1-{\mathrm{NL}}_{0}\left(t-1\right)\right){\mathrm{NL}}_{0}\left(t-1\right),$$

*with ${\mathrm{NL}}_{0}\left(0\right)\in \left[0,1\right]$ being a uniformly distributed random number. Note that, for almost all r∈[3.57,4], either the map ${\mathrm{NL}}_{0}$ is chaotic or hyperbolic roughly meaning that an attractive periodic orbit is dominating it. This is a deep result in one-dimensional dynamics (see [40] for details). In the hyperbolic case, after some transient behaviour, numerically, one only sees some periodic orbit, which has long periods in the interval r∈[3.57,4]. From the practical viewpoint, i.e., when considering short orbits, dynamics for that interval can be considered as chaotic since already small changes of r result in chaotic behaviour also in the theoretical sense.*

*Let us include some* observational noise *by adding standard white Gaussian noise ϵ to an orbit:*

$$\mathrm{NL}\left(t\right)={\mathrm{NL}}_{0}\left(t\right)+\sigma \epsilon \left(t\right),$$

*where σ>0 is the level of noise.*

Orbits of logistic maps, particularly with observational noise, are often used as a studying and illustrating tool of nonlinear time series analysis (see [41,42]). This justifies as a natural object for study a piecewise stationary noisy logistic (NL) process with change-points $t_{1}^{*},t_{2}^{*},\ldots ,t_{N_{\mathrm{st}}-1}^{*}$, defined as

$$\begin{array}{c} \mathrm{NL}\left(\left(r_{1},\ldots ,r_{N_{\mathrm{st}}}\right),\left(\sigma_{1},\ldots ,\sigma_{N_{\mathrm{st}}}\right),\left(t_{1}^{*},t_{2}^{*},\ldots ,t_{N_{\mathrm{st}}-1}^{*}\right)\right)={\left(\mathrm{NL}(t)\right)}_{t=0}^{L}, \end{array}$$

*where $r_{1},\ldots ,r_{N_{\mathrm{st}}}\in \left[3.57,4\right]$ are the values of control parameter, $\sigma_{1},\ldots ,\sigma_{N_{\mathrm{st}}}>0$ are the levels of noise, and*

$$\mathrm{NL}\left(t\right)={\mathrm{NL}}_{0}\left(t\right)+\sigma_{k}\epsilon \left(t\right),$$

*with*

$${\mathrm{NL}}_{0}\left(t\right)=r_{k}\left(1-{\mathrm{NL}}_{0}\left(t-1\right)\right){\mathrm{NL}}_{0}\left(t-1\right),$$

for all $t\in \{t_{k-1}^{*}+1,t_{k-1}^{*}+2,\ldots ,t_{k}^{*}\}$ for $k=1,2,\ldots ,N_{\mathrm{st}}$, with $t_{0}^{*}:=0$, $t_{N_{\mathrm{st}}}^{*}:=L$ and ${\mathrm{NL}}_{0}\left(0\right)\in \left[0,1\right]$ is a uniformly distributed random number.

*Figure 4b shows a realization of a ‘two-piece’ NL process with a change-point at L/2; as one can see in Figure 4d, the empirical distributions of ordinal patterns of order d=2 before the change-point and after the change-point do not coincide. In general, the distributions of ordinal patterns of order d≥1 reflect change-points for the NL processes (which can be easily checked).*

The NL and AR processes have rather different ordinal patterns distributions, being the reason for using them for empirical investigation of change-point detection methods in Section 3.

#### 2.1.3. Conditional Entropy of Ordinal Patterns

Here, we define the conditional entropy of ordinal patterns, which is a cornerstone of the suggested method for ordinal-change-point detection. Let us call a process $X={\left(X(t)\right)}_{t=0}^{L}$ for L∈N∪{∞} *ordinal-$d^{+}$-stationary* if for all $i,j\in S_{d}$ the *probability of pairs of ordinal patterns*

$$p_{i,j}=P\left(\Pi (t)=i,\Pi (t+1)=j\right)$$

does not depend on *t* for d≤t≤L−1 (compare with Definition 3). Obviously, ordinal-(d+1)-stationarity implies ordinal-$d^{+}$-stationarity.

For an ordinal-$d^{+}$-stationary stochastic process, consider the probability of an ordinal pattern $j\in S_{d}$ to occur after an ordinal pattern $i\in S_{d}$. Similarly to Equation (1), it is given by:

$$p_{j|i}=P\left(\Pi (t+1)=j|\Pi (t)=i\right)=\frac{p_{i,j}}{p_{i}}$$

for $p_{i}\neq 0$. If $p_{i}=0$, let $p_{j|i}=0$.

**Definition** **5.** *The conditional entropy of ordinal patterns of order d∈N of an ordinal $d^{+}$-stationary stochastic process X is defined by:*

(4) $$\mathrm{CE}\left(X,d\right)=-\sum_{i\in S_{d}}\sum_{j\in S_{d}}p_{i}p_{j|i}\;\ln\left(p_{i}p_{j|i}\right)+\sum_{i\in S_{d}}p_{i}\;\ln p_{i}=-\sum_{i\in S_{d}}\sum_{j\in S_{d}}p_{i}p_{j|i}\;\ln p_{j|i}.$$

For brevity, we refer to CE(X,d) as the “conditional entropy” when no confusion can arise. The conditional entropy characterizes the mean diversity of successors $j\in S_{d}$ of a given ordinal pattern $i\in S_{d}$. This quantity often provides a good practical estimation of the Kolmogorov–Sinai entropy for dynamical systems; for a discussion of this and other theoretical properties of conditional entropy, we refer to [21]. Here, we only note that the Kolmogorov–Sinai entropy quantifies unpredictability of a dynamical system.

One can estimate the conditional entropy from a time series by using the empirical conditional entropy of ordinal patterns [18]. Consider a sequence $\pi^{d,L}$ of ordinal patterns of order d∈N with length L∈N. Similarly to Equation (2), the frequency of occurrence of an ordinal patterns pair $i,j\in S_{d}$ is given by

(5) $$n_{i,j}\left(t\right)=\#\left\{l\in \left\{d,d+1,\ldots ,t-1\right\}|\pi \left(l\right)=i,\pi \left(l+1\right)=j\right\}$$

for t∈{d+1,d+2,…,L}. The *empirical conditional entropy of ordinal patterns* for $\pi^{d,L}$ is defined by

(6) $$\begin{array}{cc} \mathrm{eCE}\left(\pi^{d,L}\right)= & -\frac{1}{L-d}\sum_{i\in S_{d}}\sum_{j\in S_{d}}n_{i,j}\left(L\right)\ln n_{i,j}\left(L\right)+\frac{1}{L-d}\sum_{i\in S_{d}}n_{i}\left(L\right)\ln n_{i}\left(L\right)\\ = & -\frac{1}{L-d}\sum_{i\in S_{d}}\sum_{j\in S_{d}}n_{i,j}\left(L\right)\ln\frac{n_{i,j}\left(L\right)}{n_{i}\left(L\right)}. \end{array}$$

As a direct consequence of Lemma 1, the empirical conditional entropy approaches the conditional entropy under certain assumptions. Namely, the following holds.

**Corollary** **1.** *For the sequence $\pi^{d,\infty }$ of ordinal patterns of order d∈N of a realization of an ergodic stochastic process $X={\left(X(t)\right)}_{t\in N_{0}}$ with associated stationary increment process ${\left(X\left(t\right)-X\left(t-1\right)\right)}_{t\in N}$, it holds almost surely that*

(7) $$\lim_{L\to \infty }\mathrm{eCE}\left({\left(\pi (k)\right)}_{k=d}^{L}\right)=\mathrm{CE}\left(X,d\right).$$

### 2.2. A Statistic for Change-Point Detection Based on the Conditional Entropy of Ordinal Patterns

We now consider the classical problem of detecting a change-point $t^{*}$ on the basis of a realization *x* of a stochastic process *X* having at most one change-point, that is, it holds either $N_{\mathrm{st}}=1$ or $N_{\mathrm{st}}=2$ (compare [6]). To solve this problem, one estimates a tentative change-point ${\hat{t}}^{*}$ as the time-point that maximizes a test statistic S(t;x). Then, the value of $S({\hat{t}}^{*};x)$ is compared to a given threshold in order to decide whether ${\hat{t}}^{*}$ is a change-point.

The idea of *ordinal change-point detection* is to find change-points in a stochastic process *X* by detecting changes in the sequence $\pi^{d,L}$ of ordinal patterns for a realization of *X*. Given at most one ordinal change-point $t^{*}$ in *X*, one estimates its position ${\hat{t}}^{*}$ by using the fact that

- $\pi \left(d\right),\pi \left(d+1\right),\ldots ,\pi \left(t^{*}\right)$ characterize the process before the change;
- $\pi \left(t^{*}+1\right),\pi \left(t^{*}+2\right),\ldots ,\pi \left(t^{*}+d-1\right)$ correspond to the transitional state;
- $\pi \left(t^{*}+d\right),\pi \left(t^{*}+d+1\right),\ldots ,\pi \left(L\right)$ characterize the process after the change.

Therefore, a position of a change-point can be estimated by an ordinal-patterns-based statistic $S(t;\pi^{d,L})$ that, roughly speaking, measures dissimilarity between the distributions of ordinal patterns for ${\left(\pi (k)\right)}_{k=d}^{t}$ and for ${\left(\pi (k)\right)}_{k=t+d}^{L}$.

Then, an estimate of the change-point $t^{*}$ is given by

$${\hat{t}}^{*}=\underset{t=d,d+1,\ldots ,L}{arg max}S\left(t;\pi^{d,L}\right).$$

A method for detecting one change-point can be extended to an arbitrary number of change-points using the binary segmentation [43]: one applies a single change-point detection procedure to the realization *x*; if a change-point is detected, then it splits *x* into two segments in each of which one is looking for a change-point. This procedure is repeated iteratively for the obtained segments until all of them either do not contain change-points or are too short.

The key problem is the selection of an appropriate test statistic $S(t;\pi^{d,L})$ for detecting changes on the basis of a sequence $\pi^{d,L}$ of ordinal patterns of a realization of the process for d,L∈N. We suggest to use the following statistic:

(8) $$\begin{array}{cc} \mathrm{CEofOP}\left(t;\pi^{d,L}\right)=\left(L-2d\right)\;\mathrm{eCE}\left({\left(\pi (k)\right)}_{k=d}^{L}\right) & -\left(t-d\right)\;\mathrm{eCE}\left({\left(\pi (k)\right)}_{k=d}^{t}\right)\\ & -\left(L-(t+d)\right)\;\mathrm{eCE}\left({\left(\pi (k)\right)}_{k=t+d}^{L}\right) \end{array}$$

for all t∈N with d<t<L−d. The intuition behind this statistic comes from the concavity of conditional entropy (not only for ordinal patterns but in general, see Section 2.1.3 in [44]). It holds

(9) $$\mathrm{eCE}\left({\left(\pi (k)\right)}_{k=d}^{L}\right)\geq \frac{t-d}{L-2d}\;\mathrm{eCE}\left({\left(\pi (k)\right)}_{k=d}^{t}\right)+\frac{L-(t+d)}{L-2d}\;\mathrm{eCE}\left({\left(\pi (k)\right)}_{k=t+d}^{L}\right).$$

Therefore, if the probabilities of ordinal patterns change at some point $t^{*}$, but do not change before and after $t^{*}$, then $\mathrm{CEofOP}\left(t;\pi^{d,L}\right)$ tends to attain its maximum at $t=t^{*}$. If the probabilities do not change at all, then for *L* being sufficiently large, Inequality (9) tends to hold with equality. More rigorously, when segments of a stochastic process before and after the change-point have infinite length, the following result takes place.

**Corollary** **2.** *Let $X={\left(X_{t}\right)}_{t\in N_{0}}$ be an ergodic $d^{+}$-ordinal-stationary stochastic process on a probability space (Ω,A,P). For L∈N, let $\Pi^{d,L}$ be the random sequence of ordinal patterns of order d of $\left(X_{0},X_{1},\ldots ,X_{L}\right)$. Then, for any θ∈(0,1), it holds*

(10) $$\lim_{L\to \infty }\mathrm{CEofOP}\left(\left\lfloor \theta L\right\rfloor ;\Pi^{d,L}\right)=0,$$

*P-almost sure.*

Corollary 2 is a simple consequence of Theorem A1 (Appendix A.1). Another important property of the CEofOP statistic is its close connexion with the classical likelihood ratio statistic (see Appendix A.2 for details).

Let us now rewrite Equation (8) in a straightforward form. Let $n_{i}\left(t\right)$ and $n_{i,j}\left(t\right)$ be the frequencies of occurrence of an ordinal pattern $i\in S_{d}$ and of an ordinal patterns pair $i,j\in S_{d}$ (given by Equations (2) and (5), respectively). By setting $m_{i}\left(t\right)=n_{i}\left(L\right)-n_{i}\left(t+d\right)$ and $m_{i,j}\left(t\right)=n_{i,j}\left(L\right)-n_{i,j}\left(t+d\right)$, we get using Equation (6)

(11) $$\begin{array}{cc} \mathrm{CEofOP}\left(t;\pi^{d,L}\right)= & -\frac{L-2d}{L-d}\sum_{i\in S_{d}}\sum_{j\in S_{d}}n_{i,j}\left(L\right)\ln\frac{n_{i,j}\left(L\right)}{n_{i}\left(L\right)}\\ & +\sum_{i\in S_{d}}\sum_{j\in S_{d}}n_{i,j}\left(t\right)\ln\frac{n_{i,j}\left(t\right)}{n_{i}\left(t\right)}+\sum_{i\in S_{d}}\sum_{j\in S_{d}}m_{i,j}\left(t\right)\ln\frac{m_{i,j}\left(t\right)}{m_{i}\left(t\right)}. \end{array}$$

This statistic was first introduced and applied to the segmentation of sleep EEG time series in [18].

To demonstrate the “nonlinear” nature of the CEofOP statistic, we provide Example 5 concerning transition from a time series to its surrogate. Although being in a sense tailor-made, this example shows that CEofOP discerns changes that cannot be detected by conventional “linear” methods.

**Remark** **1.** *The question whether a time series is linear or nonlinear often arises in data analysis. For instance, linearity should be verified before using such powerful methods as Fourier analysis. For this, one usually employs a procedure known as surrogate data testing [45,46,47]. It utilises the fact that a linear time series is statistically indistinguishable from any time series sharing some of its properties (for instance, second moments and amplitude spectrum). Therefore, one can generate surrogates having the certain properties of the original time series without preserving other properties, irrelevant for a linear system. If such surrogates are significantly different from the original series, then nonlinearity is assumed.*

**Figure 5:**
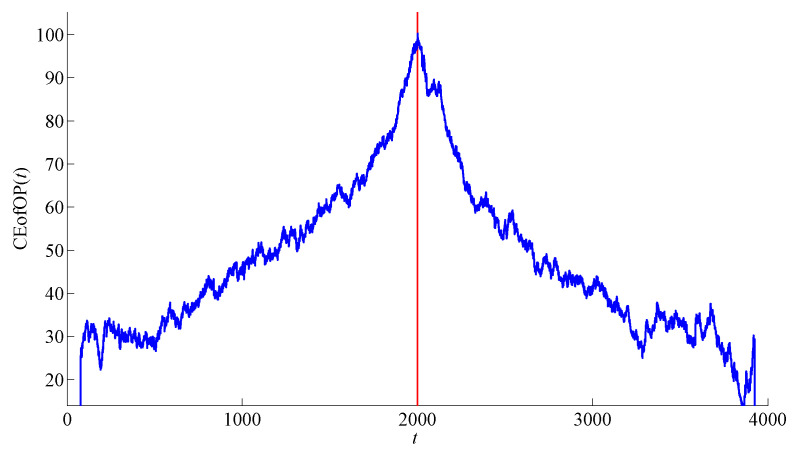
Maximum of statistic CEofOP(θL) detects the change-point $t^{*}=2000$ (indicated by the vertical line) in a time series, obtained by “gluing” a realization of a noisy logistic stochastic process $\mathrm{NL}\left(4,0.2\right)$ with its surrogate.

**Example** **5.** Consider a time series obtained by gluing a realisation of a noisy logistic process NL$\left(r,\sigma \right)$ of length L/2 (without changes) with its surrogate of the same length (to generate surrogates, we use the iterative amplitude adjusted Fourier transform (AAFT) algorithm suggested by [46] and implemented by [48]). This compound time series has a change-point at $t^{*}=L/2$, whose conventional methods may fail to detect since the surrogate has the same autocorrelation function as the original process (for instance, this is the case for the Brodsky–Darkhovsky method considered further in Section 3). However, the ordinal pattern distributions for the original time series and its surrogate generally are significantly different. Therefore, the CEofOP statistic detects the change-point, which is illustrated by Figure 5.

**Remark** **2.** *Although the idea that ordinal structure is a relevant indicator of time series linearity/nonlinearity is not new [12,15], to our knowledge, it was not rigorously proved that the distribution of ordinal patterns is altered by surrogates. This is clearly beyond the scope of this paper and will be discussed elsewhere as a separate study; here, it is sufficient for us to provide an empirical evidence for this.*

### 2.3. Algorithm for Change-Point Detection via the CEofOP Statistic

Consider a sequence $\pi^{d,L}$ of ordinal patterns of order d∈N with length L∈N, corresponding to a realization of some piecewise stationary stochastic process. To detect a single change-point via the CEofOP statistic, we first estimate its possible position by

$${\hat{t}}^{*}=\underset{t=T_{\min}+d,\ldots ,L-T_{\min}}{arg max}S\left(t;\pi^{d,L}\right),$$

where $T_{\min}$ is a minimal length of a sequence of ordinal patterns that is sufficient for a reliable estimation of empirical conditional entropy.

**Remark** **3.** *From the representation CEofOPstat, it follows that, for a reasonable computation of the CEofOP statistic, a reliable estimation of eCE before and after the assumed change-point is required. For this, the stationary parts of a process should be sufficiently long. We take $T_{\min}=\left(d+1\right)!\left(d+1\right)$, which is equal to the number of all possible pairs of ordinal patterns of order d (see [18] for details). Consequently, the length L of a time series should satisfy*

(12) $$L\gg 2T_{min}=2\left(d+1\right)!\left(d+1\right).$$

*Note that this does not impose serious limitations on the suggested method, since condition (12) is not too restrictive for d≤3. However, it implies using of either d=2 or d=3, since d=1 does not provide effective change-point detection (see Example 3 and Appendix A.1), while d>3 in most applications demands too large sample sizes.*

In order to check whether ${\hat{t}}^{*}$ is an actual change-point, we test between the hypotheses:

$H_{0}$:  parts $\pi \left(d\right),\pi \left(d+1\right),\ldots ,\pi \left({\hat{t}}^{*}\right)$ and $\pi \left({\hat{t}}^{*}+d\right),\ldots ,\pi \left(L\right)$ of the sequence $\pi^{d,L}$ come from the same distribution;
$H_{A}$:  parts $\pi \left(d\right),\pi \left(d+1\right),\ldots ,\pi \left({\hat{t}}^{*}\right)$ and $\pi \left({\hat{t}}^{*}+d\right),\ldots ,\pi \left(L\right)$ of the sequence $\pi^{d,L}$ come from different distributions.

This test is performed by comparing $\mathrm{CEofOP}({\hat{t}}^{*};\pi^{d,L})$ to a threshold *h*, such that, if the value of CEofOP is above the threshold, one rejects $H_{0}$ in favour of $H_{A}$. The choice of the threshold is ambiguous: the lower *h*, the higher the possibility of false rejection of $H_{0}$ in favour of $H_{A}$ (*false alarm*, meaning that the test indicates a change of the distribution although there is no actual change) is. On the contrary, the higher *h*, the higher the possibility of false rejection of the $H_{A}$ is.

As it is usually done, we consider the threshold *h* as a function of the desired probability α of false alarm. To compute h(α), we shuffle blocks of ordinal patterns from the original sequence, in order to create new artificial sequences. Each such sequence has the same length as the original, but the segments on the left and on the right of the assumed change-point should have roughly the same distribution of ordinal patterns, even if the original sequence is not stationary. This procedure uses the ideas described in [49,50] and is similar to block bootstrapping [51,52,53,54]. The scheme of detecting at most one change-point via the CEofOP statistic, including the computing of a threshold h(α) is provided in Algorithm 1.

**Algorithm 1** Detecting at most one change-point**Input:** sequence $\pi ={\left(\pi (k)\right)}_{k=t_{\mathrm{start}}}^{t_{\mathrm{end}}}$ of ordinal patterns of order *d*, nominal probability α of false alarm
**Output:** estimate of a change-point ${\hat{t}}^{*}$ if change-point is detected, otherwise return 0.
1:**function**DetectSingleCP(π, α)2:    $T_{\min}$ ← (d+1)!(d+1);3:    **if** $t_{\mathrm{end}}-t_{\mathrm{start}}<2T_{\min}$ **then**4:        **return** 0;           ▷ sequence is too short, no change-point can be detected5:    **end if**6:    ${\hat{t}}^{*}$ ← $\underset{t=t_{\mathrm{start}}+T_{\min},\ldots ,t_{\mathrm{end}}-T_{\min}}{arg max}\mathrm{CEofOP}\left(t;\pi \right)$;7:    $N_{\mathrm{boot}}$ ← $\left\lfloor \frac{5}{\alpha }\right\rfloor$;         ▷ number of bootstrap samples for computing threshold8:    **for** $l=1,2,\ldots ,N_{\mathrm{boot}}$ **do**           ▷ computing threshold by reshuffling9:        ξ ← randomly shuffled blocks of length (d+1) from π;10:        $c_{j}$ ← $\underset{t=t_{\mathrm{start}}+T_{\min},\ldots ,t_{\mathrm{end}}-T_{\min}}{arg max}\mathrm{CEofOP}\left(t;\xi \right)$;11:    **end for**12:    $c_{j}$ ← Sort($c_{j}$); ▷ sort the maximal values of CEofOP for bootstrap samples in decreasing order13:    *h* ← $c_{\left\lfloor \alpha N_{\mathrm{boot}}\right\rfloor }$14:    **if** $S({\hat{t}}^{*};\pi )<h$ **then**15:        **return** 0;16:    **else**17:        **return** ${\hat{t}}^{*}$;18:    **end if**19:**end function**

To detect multiple change-points, we use an algorithm that consists of two steps:

Step 1: preliminary estimation of boundaries of the stationary segments with a threshold h(2α) computed for doubled nominal probability of false alarm (that is, with a higher risk of detecting false change-points).
Step 2: verification of the boundaries and exclusion of false change-points: a change-point is searched for a merging of every two adjacent intervals.

Details of these two steps are displayed in Algorithm 2. Step 1 is the usual binary segmentation procedure as suggested in [43]. Since this procedure detects change-points sequentially, they may be estimated incorrectly. To improve localization and eliminate false change-points, we introduce Step 2 following the idea suggested in [11].

**Algorithm 2** Detecting multiple change-points**Input:** sequence $\pi ={\left(\pi (k)\right)}_{k=d}^{L}$ of ordinal patterns of order *d*, nominal probability α of false alarm.
**Output:** estimates of the number ${\hat{N}}_{\mathrm{st}}$ of stationary segments and of their boundaries ${\left({\hat{t}}_{k}^{*}\right)}_{k=0}^{{\hat{N}}_{\mathrm{st}}}$.
1:**function**DetectAllCP(π, α)2:    ${\hat{N}}_{\mathrm{st}}\leftarrow 1$; ${\hat{t}}_{0}^{*}\leftarrow 0$; ${\hat{t}}_{1}^{*}\leftarrow L$; k←0                      ▷ Step 13:    **repeat**4:        ${\hat{t}}^{*}$ ← DetectSingleCP(${\left(\pi (l)\right)}_{l={\hat{t}}_{k}^{*}+d}^{{\hat{t}}_{k+1}^{*}}$), 2α;5:        **if** ${\hat{t}}^{*}>0$ **then**6:           **Insert** ${\hat{t}}^{*}$ to the list of change-points after ${\hat{t}}_{k}^{*}$ and renumber change-points ${\hat{t}}_{k+1}^{*},\ldots ,{\hat{t}}_{{\hat{N}}_{\mathrm{st}}}^{*}$;7:           ${\hat{N}}_{\mathrm{st}}$ ← ${\hat{N}}_{\mathrm{st}}+1$;8:        **else**9:           *k* ← k+1;10:        **end if**11:    **until** $k<{\hat{N}}_{\mathrm{st}}$;12:    *k* ← 0;                                   ▷ Step 213:    **repeat**14:        ${\hat{t}}^{*}$ ← DetectSingleCP(${\left(\pi (l)\right)}_{l={\hat{t}}_{k}^{*}+d}^{{\hat{t}}_{k+2}^{*}}$, α);15:        **if** ${\hat{t}}^{*}>0$ **then**16:           ${\hat{t}}_{k+1}^{*}$ ← ${\hat{t}}^{*}$;17:           *k* ← k+1;18:        **else**19:           **Delete** ${\hat{t}}_{k+1}^{*}$ from the change-points list and renumber change-points ${\hat{t}}_{k+2}^{*},\ldots ,{\hat{t}}_{{\hat{N}}_{\mathrm{st}}}^{*}$;20:           ${\hat{N}}_{\mathrm{st}}$ ← ${\hat{N}}_{\mathrm{st}}-1$;21:        **end if**22:    **until** $k<{\hat{N}}_{\mathrm{st}}-1$;23:    **return** ${\hat{N}}_{\mathrm{st}},{\left({\hat{t}}_{k}^{*}\right)}_{k=0}^{{\hat{N}}_{\mathrm{st}}}$;24:**end function**

## 3. Numerical Simulations and Results

In this section, we empirically investigate performance of the method for change-point detection via the CEofOP statistic. We apply it to the noisy logistic processes and to autoregressive processes (see Section 2.1.2) and compare performances of change-point detection by the suggested method and by the following existing methods:

- The ordinal-patterns-based method for detecting change-points via the *CMMD statistic* [23,24]: A time series is split into windows of equal lengths W∈N, empirical probabilities of ordinal patterns are estimated in every window. If there is a ordinal change-point in the time series, then the empirical probabilities of ordinal patterns should be approximately constant before the change-point and after the change-point, but they change at the window with the change-point. To detect this change, the CMMD statistic was introduced. (Note that the definition of the CMMD statistic in [23] contains a mistake, which is corrected in [24]. The results of numerical experiments reported in [23] also do not comply with the actual definition of the CMMD statistic (see Section 4.2.1.1 and 4.5.1.1 in [22] for details). In the original papers [23,24], authors do not estimate change-points, but only the corresponding window numbers; for the algorithm of change-point estimation by means of the CMMD statistic, we refer to Section 4.5.1 in [22].
- Two versions of the *classical Brodsky–Darkhovsky method* [11]: the Brodsky–Darkhovsky method can be used for detecting changes in various characteristics of a time series $x={\left(x(t)\right)}_{t=1}^{L}$, but the characteristic of interest should be selected in advance. In this paper, we consider detecting changes in mean, which is just the basic characteristic, and in correlation function corr(x(t),x(t+1)), which reflects relations between the future and the past of a time series and seems to be a natural choice for detecting ordinal change-points. Changes in mean are detected by the generalized version of the Kolmogorov–Smirnov statistic [11]:
  $${\mathrm{BD}}^{\exp}\left(t;x,\delta \right)={\left(\frac{t(L-t)}{L^{2}}\right)}^{\delta }\;\left|\frac{1}{t}\sum_{l=1}^{t}x\left(l\right)-\frac{1}{L-t}\sum_{l=t+1}^{L}x\left(l\right)\right|,$$
  where the parameter δ∈[0,1] regulates properties of the statistic, δ=0 is basically used (see [11] for details). Changes in the correlation function are detected by the following statistic:
  $${\mathrm{BD}}^{\mathrm{corr}}\left(t;x,\delta \right)={\mathrm{BD}}^{\exp}\left(t;{\left(x(t)x(t+1)\right)}_{t=1}^{L-1},\delta \right).$$

**Remark** **4.** *Note that we consider the statistic ${\mathrm{BD}}^{\exp}$, which is intended to detect changes in mean, though ordinal-patterns-based statistics do not detect these changes. This is motivated by the fact that changes in the noisy logistic processes are on the one hand changes in mean, and, on the other hand, ordinal changes in the sense of Definition 4. Therefore, they can be detected both by ${\mathrm{BD}}^{\exp}$ and by ordinal-patterns-based statistics. In general, by the nature of ordinal time series analysis, changes in mean and in the ordinal structure are in some sense complementary.*

We use orders d=2,3,4 of ordinal patterns for computing the CEofOP statistic (d=1 provides worse results because of reduced sensitivity, while higher orders are applicable only to rather long time series due to condition (12)). For the CMMD statistic, we take d=3 and the window size W=256. There are no special reasons for this choice except the fact that W=256 is sufficient for estimating probabilities of ordinal patterns of order d=3 inside the windows, since 256>120=5(d+1)! (Section 9.3 [15]). Results of the experiments remain almost the same for 200≤W≤1000.

Nominal probability of false alarm α=0.05 has been taken for all methods (in the case of the CMMD statistic, we have used the equivalent value 0.001, see Section 4.3.2 in [22] for details).

In Section 3.1, we study how well the statistics for change-point detection estimate the position of a single change-point. Since we expect that performance of the statistics for change-point detection may strongly depend on the length of realization, we check this in Section 3.2. Finally, we investigate the performance of various statistics for detecting multiple change-points in Section 3.3.

### 3.1. Estimation of the Position of a Single Change-Point

Consider *N* = 10,000 realizations $x^{j}={\left(x^{j}\left(t\right)\right)}_{t=0}^{L}$ with j=1,…,N for each of the processes listed in Table 1. A single change occurs at a random time $t^{*}$ uniformly distributed in $\left\{\frac{L}{4}-W,\frac{L}{4}-W+1,\ldots ,\frac{L}{4}+W\right\}$. For all processes, length L=80W of sequences of ordinal patterns is taken, with W=256.

To measure the overall accuracy of change-point detection via some statistic *S* as applied to the process *X*, we use three quantities. Let us first determine the error of the change-point estimation provided by the statistic *S* for the *j*-th realization of a process *X*:

$${\mathrm{err}}^{j}\left(S,X\right)=\left({\hat{t}}^{*}\left(S;x^{j}\right)-t^{*}\right),$$

where $t^{*}$ is the actual position of the change-point and ${\hat{t}}^{*}\left(S;x^{j}\right)$ is its estimate obtained by using *S*. Then, the *fraction of satisfactorily estimated change-points* sE (averaged over *N* realizations) is defined by:

$$\mathrm{sE}\left(S,X\right)=\frac{\#\left\{j\in \left\{1,2,\ldots ,N\right\}:\;|{\mathrm{err}}^{j}\left(S,X\right)|\leq \mathrm{MaxErr}\right\}}{N},$$

where MaxErr is the maximal satisfactory error, we take MaxErr=W=256. The *bias* and the *root mean squared error* (RMSE) are respectively given by

$$\begin{array}{cc} B(S,\;X) & =\frac{1}{N}\sum_{j=1}^{N}{\mathrm{err}}^{j}\left(S,\;X\right),\\ \mathrm{RMSE}(S,\;X) & =\sqrt{\frac{1}{N}\sum_{j=1}^{N}{\left({\mathrm{err}}^{j}\left(S,\;X\right)\right)}^{2}}. \end{array}$$

A large sE and a bias and RMSE close to zero are standing for a high accuracy of the estimation of a change-point. Results of the experiments are presented in Table 2 and Table 3 for NL and AR processes, respectively. For every process, the best values of performance measures are shown in **bold**.

Let us summarize: for the considered processes, the CEofOP statistic estimates change-point more accurately than the CMMD statistic. For the NL processes, the CEofOP statistic has almost the same performance as the Brodsky–Darkhovsky method; for the AR processes, performance of the classical method is better, though CEofOP has lower bias. In contrast to the ordinal-patterns-based methods, the Brodsky–Darkhovsky method is unreliable when there is a lack of a priori information about the time series. For instance, changes in NL processes only slightly influence the correlation function and ${\mathrm{BD}}^{\mathrm{corr}}$ does not provide a good indication of changes (cf. performance of ${\mathrm{BD}}^{\mathrm{corr}}$ and CEofOP in Table 2). Here, note that level shifts before and after a time point do not change ${\mathrm{BD}}^{\mathrm{corr}}$.

Meanwhile, changes in the AR processes do not influence the expected value (see Example 3), which does not allow for detecting them using ${\mathrm{BD}}^{\exp}$ (see Table 3). Therefore, we do not consider the ${\mathrm{BD}}^{\exp}$ statistic in further experiments.

Note that performance of the CEofOP statistic is only slightly better for d=3 than for d=2, and for d=4 even decreases, although one can expect better change-point detection for higher *d*. As we show in the following session, this is due to the fact that the performance of the CEofOP statistic depends on the length *L* of the time series. In particular, L=80×256 = 20,480 is not sufficient for applying the CEofOP statistic with d=4.

### 3.2. Estimating Position of a Single Change-Point for Different Lengths of Time Series

**Figure 6:**
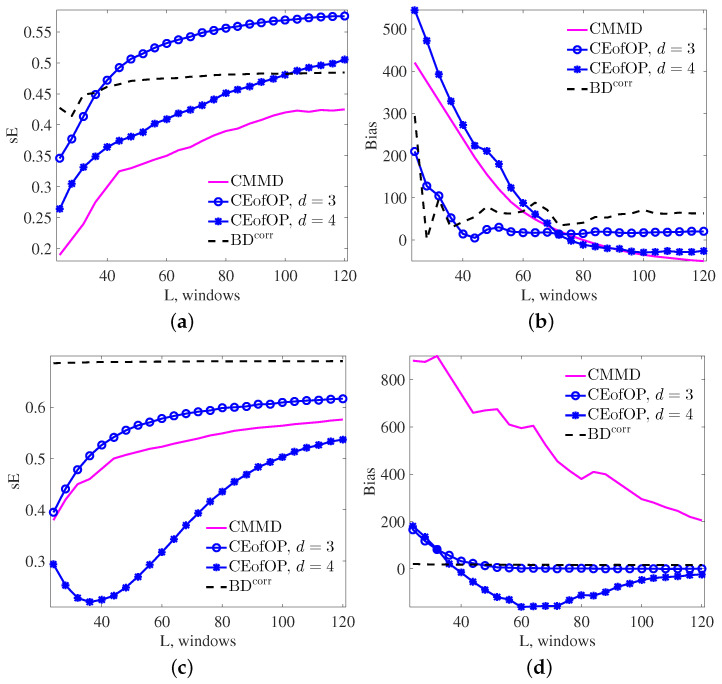
Measures of change-point detection performance for NL (**a**,**b**) and AR (**c**,**d**) processes with different lengths, where *L* is the product of window numbers given on the *x*-axis with window length W=256.

Here, we study how the accuracy of change-point estimation for the three considered statistics depends on the length *L* of a time series. We take N= 50,000 realizations of NL, 3.95→3.98, σ=0.2 and AR, 0.1→0.4 for realization lengths *L* = 24 W, 28 W, …, 120 W. Again, we consider a single change at a random time $t^{*}\in \left\{\frac{L}{4}-W,\frac{L}{4}-W+1,\ldots ,\frac{L}{4}+W\right\}$. Results of the experiment are presented in Figure 6.

In summary, performance of the CEofOP statistic is generally better than for the CMMD statistic, but strongly depends on the length of time series. This emphasizes importance of condition (12). From the results of our experiments, we recommend choosing *d*, satisfying $L>50\cdot T_{\min}=100\left(d+1\right)!\left(d+1\right)$. In comparison with the classical Brodsky–Darkhovsky method, CEofOP has better performance for NL processes (see Figure 6a,b), and lower bias for AR processes (see Figure 6d).

### 3.3. Detecting Multiple Change-Points

Here, we investigate how well the considered statistics detect multiple change-points. Methods for change-point detection via the CEofOP and the CMMD statistics are implemented according to Section 2.3 and Section 4.5.1 in [22], respectively. We consider here CEofOP only for d=3, since it provided the best change-point detection in previous experiments. The Brodsky–Darkhovsky method is implemented according to [11] with only one exception: to compute a threshold for it, we use the shuffling procedure (Algorithm 1), which in our case provided better results than the technique described in [11].

We consider here two processes, $\mathrm{AR}\left(\left(0.3,0.5,0.1,0.4\right),\left(t_{1}^{*},t_{2}^{*},t_{3}^{*}\right)\right)$ and NL((3.98,4,3.95,3.8),(0.2,0.2,0.2,0.3),$\left(t_{1}^{*},t_{2}^{*},t_{3}^{*})\right)$, with change-points $t_{k}^{*}$ being independent and uniformly distributed in $\left\{\bar{t_{k}^{*}}-W,\bar{t_{k}^{*}}-W+1,\ldots ,\bar{t_{k}^{*}}+W\right\}$ for k=1,2,3 with $\bar{t_{1}^{*}}=0.3L$, $\bar{t_{2}^{*}}=0.7L$, $\bar{t_{3}^{*}}=0.9L$, and L=100 W. For both processes, we generate N=10000 realizations $x^{j}$ with j=1,…,N. We consider unequal lengths of stationary segments to study methods for change-point detection in more realistic conditions.

As we apply change-point detection via a statistic *S* to realization $x^{j}$, we obtain estimates of the number ${\hat{N}}_{\mathrm{st}}\left(S;x^{j}\right)$ of stationary segments and of change-points positions ${\hat{t}}_{l}^{*}\left(S;x^{j}\right)$ for $l=1,2,\ldots ,{\hat{N}}_{\mathrm{st}}\left(S;x^{j}\right)-1$. Since the number of estimated change-points may be different from the actual number of changes, we suppose that the estimate for $t_{k}^{*}$ is provided by the nearest ${\hat{t}}_{l}^{*}\left(S;x^{j}\right)$. Therefore, the error of estimation of the *k*-th change-point provided by *S* is given by

$${\mathrm{err}}_{k}^{j}\left(S,X\right)=\min_{l=1,2,\ldots ,{\hat{N}}_{\mathrm{st}}\left(S;x^{j}\right)-1}|{\hat{t}}_{l}^{*}\left(S;x^{j}\right)-t_{k}^{*}|.$$

To assess the overall accuracy of change-point detection, we compute two quantities. The fraction ${\mathrm{sE}}_{k}$ of satisfactory estimates of a change-point $t_{k}^{*}$, k=1,2,3 is given by

$${\mathrm{sE}}_{k}\left(S,X\right)=\frac{\#\left\{j\in \left\{1,2,\ldots ,N\right\}|{\mathrm{err}}_{k}^{j}\left(S,X\right)\leq \mathrm{MaxErr}\right\}}{N},$$

where MaxErr is the maximal satisfactory error; we take MaxErr=W=256. The average number of *false change-points* is defined by:

$$\mathrm{fCP}\left(S,X\right)=\frac{\sum_{j=1}^{N}\;\left({\hat{N}}_{\mathrm{st}}\left(S;x^{j}\right)-1-\#\left\{k\in \left\{1,2,3\right\}|{\mathrm{err}}_{k}^{j}\left(S,X\right)\leq \mathrm{MaxErr}\right\}\right)}{N}.$$

Results of the experiment are presented in Table 4 and Table 5, and the best values are shown in **bold**.

In summary, since distributions of ordinal patterns for NL and AR processes have different properties, results for them differ significantly. The CEofOP statistic provides good results for the NL processes. However, for the AR processes, its performance is much worse: only the most prominent change is detected rather well. Weak results for two other change-points are caused by the fact that the CEofOP statistic is rather sensitive to the lengths of stationary segments (we have already seen this in Section 3.2), and in this case they are not very long.

## 4. Conclusions and Open Points

In this paper, we have introduced a method for change-point detection via the CEofOP statistic and have tested it for time series coming from two classes of models with quite different behavior, namely piecewise stationary noisy logistic and autoregressive processes.

The empirical investigations suggest that the method proposed provides better detection of ordinal change-points than the ordinal-patterns-based method introduced in [23,24]. Performance of our method for the two model classes considered is particularly comparable to that for the classical Brodsky–Darkhovsky method, but, in contrast to it, ordinal-patterns-based methods require less a priori knowledge about the time series. This can be especially useful in the case of considering nonlinear models where the autocorrelation function does not describe distributions completely. Here, the point is that with exception of the mean much of the distribution is captured by its ordinal structure. Thus (together with methods finding changes in mean), the CEofOP statistic can be used at least for a first exploration step. It is remarkable that our method behaves well with respect to the bias of the estimation, possibly qualifying it to improve localization of change-points found by other methods.

Although numerical experiments and tests to real-world data cannot replace rigorous theoretical studies, the results of the current study show the potential of the change-point detection via the CEofOP statistic. However, there are some open points listed below:

1. A method for computing a threshold *h* for the CEofOP statistic without shuffling the original time series is of interest, since this procedure is rather time consuming. One possible solution is to utilize Theorem A1 (Appendix A.1) and to precompute thresholds using the values of $\Delta_{\gamma ,\theta }^{d}\left(P,Q\right)$. However, this approach requires further investigation.
2. The binary segmentation procedure [43] is not the only possible method for detecting multiple change-points. In [8,55], an alternative approach is suggested: the number of stationary segments ${\hat{N}}_{\mathrm{st}}$ is estimated by optimizing a contrast function, then the positions of the change-points are adjusted. Likewise, one can consider a method for multiple change-point detection based on maximizing the following generalization of CEofOP statistic:
  $$\mathrm{CEofOP}\left(t\right)=\left(L-d{\hat{N}}_{\mathrm{st}}\right)\;\mathrm{eCE}\left({\left(\pi (k)\right)}_{k=d}^{L}\right)-\sum_{l=1}^{{\hat{N}}_{\mathrm{st}}}\left({\hat{t}}_{l}^{*}-{\hat{t}}_{l-1}^{*}-d\right)\;\mathrm{eCE}\left(\pi \left({\hat{t}}_{l-1}^{*}+d\right),\ldots ,\pi \left({\hat{t}}_{l}^{*}\right)\right),$$
  where ${\hat{N}}_{\mathrm{st}}\in N$ is an estimate of number of stationary segments, ${\hat{t}}_{1}^{*}=0$, ${\hat{t}}_{{\hat{N}}_{\mathrm{st}}}^{*}=L$ and ${\hat{t}}_{1}^{*},{\hat{t}}_{2}^{*},\ldots ,{\hat{t}}_{{\hat{N}}_{\mathrm{st}}-1}^{*}\in N$ are estimates of change-points. Further investigation in this direction could be of interest.
3. As we have seen in Section 3.2, CEofOP statistic requires rather large sample sizes to provide reliable change-point detection. This is due to the necessity of the empirical conditional entropy estimation (see Section 2.3). In order to reduce the required sample size, one may consider more effective estimates of the conditional entropy—for instance, the Grassberger estimate (see [56] and also Section 3.4.1 in [22]). However, elaboration of this idea is beyond the scope of this paper.
4. We did not use the full power of ordinal time series analysis, which often considers ordinal patterns taken from sequences of equidistant time points of some distance τ. This generalization of the case τ=1 with successive points allows for addressing different scales and so to extract more information on the distribution of a time series [57], also being useful for change-point detection.
5. In this paper, only one-dimensional time series are considered, though there is no principal limitation for applying ordinal-patterns-based methods to multivariate data (see [28]). Discussion of using ordinal-patterns-based methods for detecting change-point in multivariate data (for instance, in multichannel EEG) is therefore of interest.
6. We have considered here only the “offline” detection of changes, which is used when the acquisition of a time series is completed. Meanwhile, in many applications, it is necessary to detect change-points “online”, based on a small number of observations after the change [1]. Development of online versions of ordinal-patterns-based methods for change-point detection may be an interesting direction of a future work.

## Figures and Tables

**Table 1:** Processes used for investigation of the change-point detection.

Short Name	Complete Designation
NL, 3.95→3.98, σ=0.2	$\mathrm{NL}\left(\left(3.95,3.98\right),\left(0.2,0.2\right),t^{*}\right)$
NL, 3.95→3.80, σ=0.3	$\mathrm{NL}\left(\left(3.95,3.80\right),\left(0.3,0.3\right),t^{*}\right)$
NL, 3.95→4.00, σ=0.2	$\mathrm{NL}\left(\left(3.95,4.00\right),\left(0.2,0.2\right),t^{*}\right)$
AR, 0.1→0.3	$\mathrm{AR}\left(\left(0.1,0.3\right),t^{*}\right)$
AR, 0.1→0.4	$\mathrm{AR}\left(\left(0.1,0.4\right),t^{*}\right)$
AR, 0.1→0.5	$\mathrm{AR}\left(\left(0.1,0.5\right),t^{*}\right)$

**Table 2:** Performance of different statistics for estimating change-point in noisy logistic (NL) processes

	NL, 3.95→3.98			NL, 3.95→3.80			NL, 3.95→4.00		
Statistic	σ=0.2			σ=0.3			σ=0.2		
	sE	B	RMSE	sE	B	RMSE	sE	B	RMSE
CMMD	0.34	698	1653	0.50	−51	306	0.68	−13	206
CEofOP,d=2	0.46	147	1108	0.62	−3	267	0.81	33	147
CEofOP,d=3	0.61	53	397	0.65	**1**	256	0.88	20	99
CEofOP,d=4	0.47	**−2**	982	0.46	−41	1162	0.83	2	130
${\mathrm{BD}}^{\exp}$	**0.62**	78	**351**	**0.78**	−6	**145**	**0.89**	43	**96**
${\mathrm{BD}}^{\mathrm{corr}}$	0.44	85	656	0.71	13	202	0.77	43	189

**Table 3:** Performance of different statistics for estimating change-point in autoregressive (AR) processes.

Statistic	AR, 0.1→0.3			AR, 0.1→0.4			AR, 0.1→0.5		
	sE	B	RMSE	sE	B	RMSE	sE	B	RMSE
CMMD	0.32	616	1626	0.54	−14	368	0.68	−48	184
CEofOP,d=2	0.42	74	1096	0.67	6	244	0.82	3	129
CEofOP,d=3	0.39	126	1838	0.68	**0**	234	0.86	**0**	110
CEofOP,d=4	0.08	1028	6623	0.46	−176	1678	0.74	−27	214
${\mathrm{BD}}^{\exp}$	0.00	>$10^{3}$	>$10^{4}$	0.00	>$10^{4}$	>$10^{4}$	0.00	>$10^{4}$	>$10^{4}$
${\mathrm{BD}}^{\mathrm{corr}}$	**0.79**	**31**	**151**	**0.92**	21	**73**	**0.97**	21	**50**

**Table 4:** Performance of change-point detection methods for the process with three change-points $\mathrm{NL}\left(\left(3.98,4,3.95,3.8\right),\left(0.2,0.2,0.2,0.3\right),\left(t_{1}^{*},t_{2}^{*},t_{3}^{*}\right)\right)$.

Statistic	Number of False Change-Points	Fraction ${\mathrm{sE}}_{k}$ of Satisfactory Estimates			
		1st Change	2nd Change	3rd Change	Average
cMMD	1.17	0.465	0.642	0.747	0.618
CEofOP	**0.62**	**0.753**	**0.882**	**0.930**	**0.855**
${\mathrm{BD}}^{\mathrm{corr}}$	1.34	0.296	0.737	0.751	0.595

**Table 5:** Performance of change-point detection methods for the process with three change-points $\mathrm{AR}\left(\left(0.3,0.5,0.1,0.4\right),\left(t_{1}^{*},t_{2}^{*},t_{3}^{*}\right)\right)$.

Statistic	Number of False Change-Points	Fraction ${\mathrm{sE}}_{k}$ of Satisfactory Estimates			
		1st Change	2nd Change	3rd Change	Average
CMMD	1.17	0.340	0.640	0.334	0.438
CEofOP	1.12	0.368	0.834	0.517	0.573
${\mathrm{BD}}^{\mathrm{corr}}$	**0.53**	**0.783**	**0.970**	**0.931**	**0.895**

**Figure A1:**
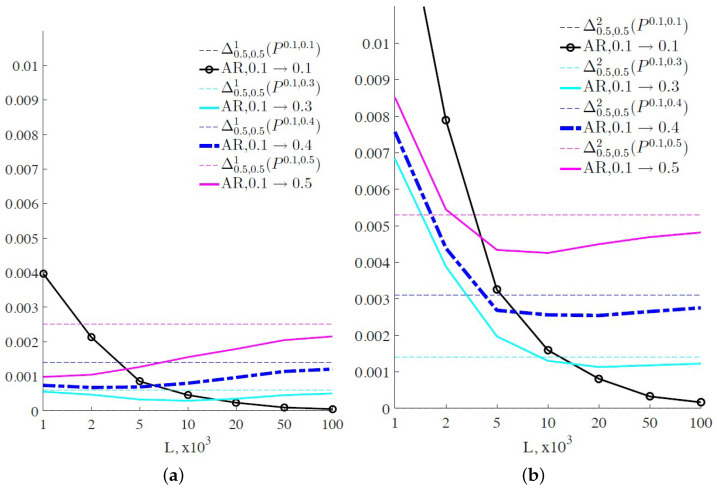
Empirical values of CEofOP statistics $\frac{1}{L}\max_{\theta \in (0,1)}\mathrm{CEofOP}\left(\left\lfloor \theta L\right\rfloor ;\pi^{d,L}\right)$ converge to the theoretical values $\Delta_{0.5,0.5}^{d}$ for autoregressive processes as *L* increases for d=1 (**a**) and d=2 (**b**). Here, AR, $\phi_{1}\to \phi_{2}$ stands for the process $\mathrm{AR}\left((\phi_{1},\phi_{2}),L/2\right)$. The provided empirical values are obtained either as 5th percentile (for $\phi_{1}\neq \phi_{2}$) or as 95th percentile (for $\phi_{1}=\phi_{2}$) from 1000 trials.

**Figure A2:**
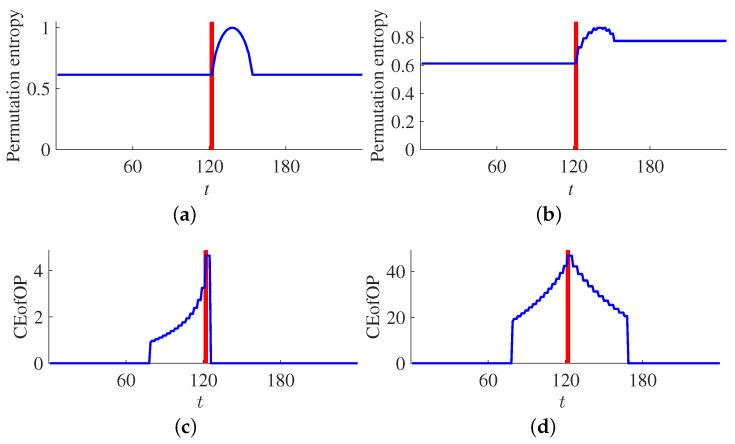
Permutation entropy computed in sliding windows (**a**,**b**) and values of the CEofOP statistics (**c**,**d**) for artificial time series with sequences of ordinal patterns $\pi_{1}^{d,L}$ and $\pi_{2}^{d,L}$, respectively, where d=3, L=240. Both sequences of ordinal patterns have a change-point at t=L/2 (indicated by red vertical line) and are given by $\pi_{1}^{d,L}=\left(3,5,1\ldots ,3,5,1,0,4,2,0,4,2,\ldots ,0,4,2\right)$ and $\pi_{2}^{d,L}=\left(3,5,1\ldots ,3,5,1,0,4,5,1,0,4,5,1,\ldots ,0,4,5,1\right)$. Permutation entropy is computed in sliding windows of length 5(d+1)!=30. While peaks of the CEofOP statistics clearly indicate the change-points, there is no straightforward way to detect changes from the values of permutation entropy.

## Appendix A. Theoretical Underpinnings of the CEofOP Statistic

### Appendix A.1. Asymptotic Behavior of the CEofOP Statistic

Here, we consider the values of CEofOP for the case when segments of a stochastic process before and after the change-point have infinite length.

Let us first introduce some notation. Given an ordinal-$d^{+}$-stationary stochastic process *X* for d∈N, the distribution of pairs of ordinal patterns is denoted by $P={\left(p_{i,j}\right)}_{i,j\in S_{d}}$, with $p_{i,j}=P(\Pi \left(t\right)=i,$$\Pi \left(t+1\right)=j)=p_{j|i}p_{i}$ for all $i,j\in S_{d}$. One easily sees the following: the conditional entropy of ordinal patterns is represented as CE(X,d)=H(P), where

$$H\left(P\right)=-\sum_{i\in S_{d}}\sum_{j\in S_{d}}p_{i,j}\ln p_{i,j}+\sum_{i\in S_{d}}\left(\sum_{j\in S_{d}}p_{i,j}\right)\ln\sum_{j\in S_{d}}p_{i,j}.$$

Here, recall that $p_{i}=\sum_{j\in S_{d}}p_{i,j}$.

**Theorem** **A1.**
*Let $Y={\left(Y_{t}\right)}_{t\in N}$ and $Z={\left(Z_{t}\right)}_{t\in N}$ be ergodic $d^{+}$-ordinal-stationary stochastic processes on a probability space (Ω,A,P) with probabilities of pairs of ordinal patterns of order d∈N given by $P={\left(p_{i,j}\right)}_{i,j\in S_{d}}$ and $Q={\left(q_{i,j}\right)}_{i,j\in S_{d}}$, respectively. For L∈N and γ∈(0,1), let $\Pi_{L,\gamma }$ be the random sequence of ordinal patterns of order d of*

(A1) $$(Y_{1},\ldots ,Y_{\left\lfloor \gamma L\right\rfloor },Z_{\lfloor \gamma L\rfloor +1},Z_{\lfloor \gamma L\rfloor +2},\ldots ,Z_{L}).$$

*Then, for all θ∈(0,1), it holds that*

$$\lim_{L\to \infty }\mathrm{CEofOP}\left(\left\lfloor \theta L\right\rfloor ;\Pi_{L,\gamma }\right)=L\;\Delta_{\gamma ,\theta }^{d}\left(P,Q\right),$$

*P-almost sure, where*

$$\Delta_{\gamma ,\theta }^{d}\left(P,Q\right)\;=\;\left\{\begin{array}{cc} \;H\left(\gamma P+(1\;-\;\gamma )Q\right)-\theta H\left(P\right)-\left(1\;-\;\theta \right)H\left(\frac{\gamma -\theta }{1-\theta }P+\frac{1-\gamma }{1-\theta }Q\right), & \;\;\theta <\gamma ,\\ \;H\left(\gamma P+(1\;-\;\gamma )Q\right)-\theta H\left(\frac{\gamma }{\theta }P+\frac{\theta -\gamma }{\theta }Q\right)-\left(1\;-\;\theta \right)H\left(Q\right), & \;\;\theta \geq \gamma . \end{array}\right.$$

By definition, Equation (A1) defines a stochastic process of length L+1 with a potential ordinal change-point $t^{*}=\left\lfloor \theta L\right\rfloor$, i.e., the position of $t^{*}$ relative to *L* is principally the same for all *L*, and the statistics considered are stabilizing for increasing *L*. Equation (A1) can be particularly interpreted as a part of a stochastic process including exactly one ordinal chance point. We omit the proof of Theorem A1 since it is a simple computation.

Due to the properties of the conditional entropy, it holds that

$$\max_{\theta \in (0,1)}\Delta_{\gamma ,\theta }^{d}\left(P,Q\right)=\Delta_{\gamma ,\gamma }^{d}\left(P,Q\right)=H\left(\gamma P+(1-\gamma )Q\right)-\gamma H\left(P\right)-\left(1-\gamma \right)H\left(Q\right).$$

Values of $\Delta_{\gamma ,\theta }^{d}\left(P,Q\right)$ can be computed for a piecewise stationary stochastic process with known probabilities of ordinal patterns before and after the change-point. To apply Theorem A1, probabilities of pairs of ordinal patterns of order *d* are also needed, but they can be calculated from the probabilities of ordinal patterns of order (d+1). As one can verify, probability $P_{i,j}$ of any pair (i,j) of ordinal patterns $i,j\in S_{d}$ is equal either to the probability of a certain ordinal pattern of order (d+1) or to the sum of two such probabilities.

In [13], authors compute probabilities of ordinal patterns of orders d=2 (Proposition 5.3) and d=3 (Theorem 5.5) for stationary Gaussian processes (in particular, for autoregressive processes). Below, we use these results to illustrate Theorem A1.

Consider an autoregressive process $\mathrm{AR}\left(\left(\phi_{1},\phi_{2}\right),t^{*}\right)$ with a single change-point $t^{*}=L/2$ for L∈N. Using the results from [13], we compute distributions $P^{\phi_{1}}$, $P^{\phi_{2}}$ of ordinal pattern pairs for orders d=1,2 and, on this basis, we calculate the values of $\Delta_{0.5,0.5}^{d}\left(P^{\phi_{1}},P^{\phi_{2}}\right)$ for different values of $\phi_{1}$ and $\phi_{2}$. The results are presented in Table A1 and Table A2.

**Table A1:** Values of $100\;\Delta_{0.5,0.5}^{1}\left(P^{\phi_{1}},P^{\phi_{2}}\right)$ for an autoregressive process (coefficient 100 here is only for the sake of readability).

	$\phi_{1}$	0.00	0.10	0.20	0.30	0.40	0.50	0.60	0.70	0.80	0.90	0.99
$\phi_{2}$												
0.00		0	0.02	0.07	0.15	0.26	0.40	0.56	0.74	0.95	1.18	1.44
0.10		0.02	0	0.02	0.06	0.14	0.25	0.37	0.53	0.71	0.91	1.13
0.20		0.07	0.02	0	0.02	0.06	0.13	0.23	0.36	0.51	0.68	0.88
0.30		0.15	0.06	0.02	0	0.01	0.06	0.13	0.22	0.34	0.49	0.66
0.40		0.26	0.14	0.06	0.01	0	0.01	0.06	0.12	0.22	0.33	0.48
0.50		0.40	0.25	0.13	0.06	0.01	0	0.01	0.05	0.12	0.21	0.33
0.60		0.56	0.37	0.23	0.13	0.06	0.01	0	0.01	0.05	0.12	0.21
0.70		0.74	0.53	0.36	0.22	0.12	0.05	0.01	0	0.01	0.05	0.12
0.80		0.95	0.71	0.51	0.34	0.22	0.12	0.05	0.01	0	0.01	0.05
0.90		1.18	0.91	0.68	0.49	0.33	0.21	0.12	0.05	0.01	0	0.01
0.99		1.44	1.13	0.88	0.66	0.48	0.33	0.21	0.12	0.05	0.01	0

**Table A2:** Values of $100\;\Delta_{0.5,0.5}^{2}\left(P^{\phi_{1}},P^{\phi_{2}}\right)$ for an autoregressive process.

	$\phi_{1}$	0.00	0.10	0.20	0.30	0.40	0.50	0.60	0.70	0.80	0.90	0.99
$\phi_{2}$												
0.00		0	0.04	0.15	0.33	0.56	0.85	1.18	1.55	1.95	2.40	2.88
0.10		0.04	0	0.04	0.14	0.31	0.53	0.80	1.12	1.48	1.89	2.34
0.20		0.15	0.04	0	0.03	0.13	0.29	0.51	0.77	1.08	1.44	1.85
0.30		0.33	0.14	0.03	0	0.03	0.13	0.28	0.49	0.75	1.06	1.43
0.40		0.56	0.31	0.13	0.03	0	0.03	0.12	0.27	0.48	0.74	1.06
0.50		0.85	0.53	0.29	0.13	0.03	0	0.03	0.12	0.27	0.48	0.74
0.60		1.18	0.80	0.51	0.28	0.12	0.03	0	0.03	0.12	0.27	0.48
0.70		1.55	1.12	0.77	0.49	0.27	0.12	0.03	0	0.03	0.12	0.28
0.80		1.95	1.48	1.08	0.75	0.48	0.27	0.12	0.03	0	0.03	0.13
0.90		2.40	1.89	1.44	1.06	0.74	0.48	0.27	0.12	0.03	0	0.03
0.99		2.88	2.34	1.85	1.43	1.06	0.74	0.48	0.28	0.13	0.03	0

According to Theorem A1, for $\pi^{d,L}={\left(\pi (k)\right)}_{k=d}^{L}$, being a sequence of ordinal patterns of order *d* for a realization of $\mathrm{AR}\left((\phi_{1},\phi_{2}),L/2\right)$, it holds almost certainly that

$$\frac{1}{L}\max_{\theta \in (0,1)}\mathrm{CEofOP}\left(\left\lfloor \theta L\right\rfloor ;\pi^{d,L}\right)\underset{L\to \infty }{\to }\Delta_{0.5,0.5}^{d}\left(P^{\phi_{1}},P^{\phi_{2}}\right).$$

Figure A1 shows how fast that this convergence is. Note that the CEofOP statistic for orders d=1,2 allows for distinguishing between change and no change in the considered processes for $L\geq 20\times 10^{3}$. For $L=10^{5}$, the values of the CEofOP statistic for order d=2 are already very close to its theoretical values, whereas, for d=1, this length does not seem sufficient.

### Appendix A.2. CEofOP Statistic for a Sequence of Ordinal Patterns Forming a Markov Chain

In this subsection, we show that there is a connection between the CEofOP statistic and the classical likelihood ratio statistic. Though taking place only in a particular case, this connection reveals the nature of the CEofOP statistic.

First, we set up necessary notations. Consider a sequence $\pi^{d,L}$ of ordinal patterns for that transition probabilities of ordinal patterns may change at some t∈{d,d+1…,L}. The basic statistic for testing whether there is a change in the transition probabilities is the likelihood ratio statistic ([1], Section 2.2.3):

(A2) $$\mathrm{LR}\left(t;\pi^{d,L}\right)=-2\ln\mathrm{Lkl}\left(H_{0}|\pi^{d,L}\right)+2\ln\mathrm{Lkl}\left(H_{A}|\pi^{d,L}\right),$$

where $\mathrm{Lkl}\left(H|\pi^{d,L}\right)$ is the likelihood of the hypothesis *H* given a sequence $\pi^{d,L}$ of ordinal patterns, and the hypotheses are given by

$$\begin{array}{cc} H_{0} & :{\left(p_{j|i}\left(t\right)\right)}_{i,j\in S_{d}}={\left(q_{j|i}\left(t\right)\right)}_{i,j\in S_{d}},\\ H_{A} & :{\left(p_{j|i}\left(t\right)\right)}_{i,j\in S_{d}}\neq {\left(q_{j|i}\left(t\right)\right)}_{i,j\in S_{d}}, \end{array}$$

where $p_{j|i}\left(t\right)$, $q_{j|i}\left(t\right)$ are transition probabilities of ordinal patterns before and after *t*, respectively.

**Proposition** **A1.**
*Random sequence $\Pi^{d}$ of ordinal patterns of order d∈N forms a Markov chain with at most one change-point; then, for a sequence $\pi^{d,L}={\left(\pi (k)\right)}_{k=d}^{L}$ of ordinal patterns being a realization of $\Pi^{d}$ of length L∈N, it holds that*

$$\mathrm{LR}\left(t;\pi^{d,L}\right)=2\;\mathrm{CEofOP}\left(t;\pi^{d,L}\right)+2d\cdot \mathrm{eCE}\left(\pi^{d,L}\right).$$

**Proof.** First, we estimate the probabilities and the transition probabilities before (*p*) and after (*q*) the change ([58], Section 2):

$$\begin{array}{cc} {\hat{p}}_{i}\left(t\right) & =\frac{n_{i}\left(t\right)}{t-d},\;\;\;\;\;\;\;\;\;\;\;\;\;\;\;\;\;\;\;\;\;\;\;\;{\hat{p}}_{j|i}\left(t\right)=\frac{n_{i,j}\left(t\right)}{n_{i}\left(t\right)},\\ {\hat{q}}_{i}\left(t\right) & =\frac{m_{i}\left(t\right)}{L-(t+d)},\;\;\;\;\;\;\;\;{\hat{q}}_{j|i}\left(t\right)=\frac{m_{i,j}\left(t\right)}{m_{i}\left(t\right)}. \end{array}$$

Then, as one can see from ([58], Section 3.2), we have

$$\begin{array}{cc} \mathrm{Lkl}\left(H_{0}|\pi^{d,L}\right) & ={\hat{p}}_{\pi (d)}\left(L\right)\prod_{l=d}^{L-1}{\hat{p}}_{\pi (l+1)|\pi (l)}\left(L\right)={\hat{p}}_{\pi (d)}\left(L\right)\prod_{i\in S_{d}}\prod_{j\in S_{d}}{\left({\hat{p}}_{j|i}\left(L\right)\right)}^{n_{i,j}\left(L\right)},\\ \mathrm{Lkl}\left(H_{A}|\pi^{d,L}\right) & ={\hat{p}}_{\pi (d)}\left(t\right)\prod_{l=d}^{t}{\hat{p}}_{\pi (l+1)|\pi (l)}\left(t\right)\prod_{l=t+d}^{L-1}{\hat{q}}_{\pi (l+1)|\pi (l)}\left(t\right)\\ & ={\hat{p}}_{\pi (d)}\left(t\right)\prod_{i\in S_{d}}\prod_{j\in S_{d}}{\left({\hat{p}}_{j|i}\left(t\right)\right)}^{n_{i,j}\left(t\right)}\prod_{i\in S_{d}}\prod_{j\in S_{d}}{\left({\hat{q}}_{j|i}\left(t\right)\right)}^{m_{i,j}\left(t\right)}. \end{array}$$

Assume that the first ordinal pattern π(d) is fixed in order to simplify the computations. Then, ${\hat{p}}_{\pi (d)}\left(L\right)={\hat{p}}_{\pi (d)}\left(t\right)$ and it holds that:

$$\begin{array}{cc} \mathrm{LR}\left(t;\pi^{d,L}\right)=-2\sum_{i\in S_{d}}\sum_{j\in S_{d}}n_{i,j}\left(L\right)\left(\ln n_{i,j}\left(L\right)-\ln n_{i}\left(L\right)\right)+ & 2\sum_{i\in S_{d}}\sum_{j\in S_{d}}n_{i,j}\left(t\right)\left(\ln n_{i,j}\left(t\right)-\ln n_{i}\left(t\right)\right)\\ + & 2\sum_{i\in S_{d}}\sum_{j\in S_{d}}m_{i,j}\left(t\right)\left(\ln m_{i,j}\left(t\right)-\ln m_{i}\left(t\right)\right). \end{array}$$

Since $\sum_{j\in S_{d}}n_{i,j}\left(t\right)=n_{i}\left(t\right)$, one finally obtains:

$$\begin{array}{cc} \mathrm{LR}\left(t;\pi^{d,L}\right)=2\left(L-d\right)\;\mathrm{eCE}\left({\left(\pi (k)\right)}_{k=d}^{L}\right) & -2\left(t-d\right)\;\mathrm{eCE}\left({\left(\pi (k)\right)}_{k=d}^{t}\right)\\ & -2\left(L-(t+d)\right)\;\mathrm{eCE}\left({\left(\pi (k)\right)}_{k=t+d}^{L}\right)\\ = & 2\;\mathrm{CEofOP}\left(t;\pi^{d,L}\right)+2d\cdot \mathrm{eCE}\left(\pi^{d,L}\right).\;☐ \end{array}$$

### Appendix A.3. Change-Point Detection by the CEofOP Statistic and from Permutation Entropy Values

One may ask whether special techniques of ordinal change-point detection make sense at all when one can simply compute permutation entropy [12] of a time series in sliding windows and then apply traditional methods for change-point detection to the resulting sequence of permutation entropy values. Indeed, permutation entropy that measures non-uniformity of ordinal patterns distribution is sensitive to the changes in this distribution and can be an indicator of ordinal change-points. However, we show in Figure A2, there is no straightforward way to detect change-point from permutation entropy values.
